# Carbon dioxide is a triple vasodilator

**DOI:** 10.1093/cvr/cvag134

**Published:** 2026-06-23

**Authors:** Dragos A Duse, Nathalie H Schröder, Sotirios Akritidis, Julia Kälsch, Dorothe Möllmann, Martin Schlattjan, Julia Hoppe, Evanthia Mergia, Martin Lainka, Kurt W Schmid, Sylvia Hartmann, Christoph M Schaefer, Finn Wichmann, Amin Polzin, Ralf Erkens, Christian Jung, Malte Kelm, Bodo Levkau, Hideo A Baba

**Affiliations:** Institute for Molecular Medicine III, University Hospital Düsseldorf, Heinrich Heine University, Universitätsstr. 1, 40225 Düsseldorf, Germany; Department of Cardiology, Pulmonology, and Vascular Medicine, Medical Faculty, Heinrich Heine University, Moorenstraße 5, 40225 Düsseldorf, Germany; Institute for Molecular Medicine III, University Hospital Düsseldorf, Heinrich Heine University, Universitätsstr. 1, 40225 Düsseldorf, Germany; Division of Vascular and Endovascular Surgery, Department of General, Visceral, Vascular and Transplantation Surgery, University of Duisburg-Essen, Hufelandstraße 55, 45147 Essen, Germany; Department of Gastroenterology and Hepatology, University Duisburg-Essen, Hufelandstraße 55, 45147 Essen, Germany; Institute of Pathology, University of Duisburg-Essen, Hufelandstraße 55, 45147 Essen, Germany; Institute of Pathology, University of Duisburg-Essen, Hufelandstraße 55, 45147 Essen, Germany; Institute for Molecular Medicine III, University Hospital Düsseldorf, Heinrich Heine University, Universitätsstr. 1, 40225 Düsseldorf, Germany; Institute of Pharmacology and Toxicology, Ruhr-University Bochum, Universitätsstrasse 150, 44780 Bochum, Germany; Division of Vascular and Endovascular Surgery, Department of General, Visceral, Vascular and Transplantation Surgery, University of Duisburg-Essen, Hufelandstraße 55, 45147 Essen, Germany; Institute of Pathology, University of Duisburg-Essen, Hufelandstraße 55, 45147 Essen, Germany; Institute of Pathology, University of Duisburg-Essen, Hufelandstraße 55, 45147 Essen, Germany; Institute of Pathology, University of Duisburg-Essen, Hufelandstraße 55, 45147 Essen, Germany; Institute of Pathology, University of Duisburg-Essen, Hufelandstraße 55, 45147 Essen, Germany; Department of Cardiology, Pulmonology, and Vascular Medicine, Medical Faculty, Heinrich Heine University, Moorenstraße 5, 40225 Düsseldorf, Germany; CARID - Cardiovascular Research Institute Düsseldorf, Moorenstraße 5, 40225 Düsseldorf, Germany; Department of Cardiology, Pulmonology, and Vascular Medicine, Medical Faculty, Heinrich Heine University, Moorenstraße 5, 40225 Düsseldorf, Germany; Department of Cardiology, Pulmonology, and Vascular Medicine, Medical Faculty, Heinrich Heine University, Moorenstraße 5, 40225 Düsseldorf, Germany; CARID - Cardiovascular Research Institute Düsseldorf, Moorenstraße 5, 40225 Düsseldorf, Germany; Department of Cardiology, Pulmonology, and Vascular Medicine, Medical Faculty, Heinrich Heine University, Moorenstraße 5, 40225 Düsseldorf, Germany; CARID - Cardiovascular Research Institute Düsseldorf, Moorenstraße 5, 40225 Düsseldorf, Germany; Institute for Molecular Medicine III, University Hospital Düsseldorf, Heinrich Heine University, Universitätsstr. 1, 40225 Düsseldorf, Germany; CARID - Cardiovascular Research Institute Düsseldorf, Moorenstraße 5, 40225 Düsseldorf, Germany; Institute of Pathology, University of Duisburg-Essen, Hufelandstraße 55, 45147 Essen, Germany

**Keywords:** Carbon dioxide, Near-infrared spectroscopy, Nitric oxide, Potassium channels, Endothelium-derived hyperpolarization, Carbonic anhydrase, Peripheral artery disease

## Abstract

**Aims:**

Carbon dioxide (CO_2_) can regulate blood flow and is applied therapeutically in intensive care units to treat brain injury, as well as in balneotherapy for peripheral arterial disease (PAD) and diabetic angiopathy; however, its mode of action remains unclear.

**Methods and results:**

The vasoactive CO_2_ effects were tested in arteries of healthy C57BL/6J mice, hypertensive apolipoprotein E-deficient mice, and soluble guanylyl cyclase (sGC) knockout mice in a small vessel myograph with and without pharmacologically intervening in endothelium- and/or vascular smooth muscle-mediated vasodilation. CO_2_-based near-infrared spectroscopy (NIRS-CO_2_) was developed to assess vasoreactivity of the skin microcirculation to CO_2_ in healthy individuals, PAD and coronary artery disease (CAD) patients, and was compared with flow-mediated dilation (FMD). We identified CO_2_ as a triple vasodilator mimicking the actions of endothelium-derived relaxing factor (nitric oxide, NO), endothelium-derived hyperpolarization factor, and direct myogenic vasodilators. CO_2_ engaged endothelial NO/sGC, endothelial small-/intermediate-conductance calcium-activated potassium channels (SK_Ca_/IK_Ca_), and myogenic voltage-gated (K_V_) and IK_Ca_ potassium channels, respectively, acting as a triple vasodilator. CO_2_-evoked vasodilator responses were blunted and delayed in diseased human and murine arteries. In the human cohort, the NIRS-CO_2_-derived time-to-intersection of the HbO_2_ and HHb curves, capturing the delay phenotype, showed a strong association with PAD/CAD status and, in exploratory analyses, also distinguished young individuals with cardiovascular risk factors, supporting NIRS-CO_2_ as a physiological readout that integrates endothelial and myogenic components of microvascular reactivity. Duration and extent of CO_2_ vasodilation were coupled to tissue metabolism through vascular carbonic anhydrases (CAs), providing a mechanism for vasculometabolic coupling and one for clinically approved CA inhibitors.

**Conclusion:**

NIRS-CO_2_ provides a feasible readout of CO_2_-evoked microvascular responsiveness and shows disease-associated alterations in our PAD/CAD cohort. Larger studies will validate generalizability across vasculopathies and clarify the relative contributions of NO-sGC vs. K^+^ channel-linked mechanisms for future therapeutic translation.

## Graphical Abstract

**Graphical Abstract:**
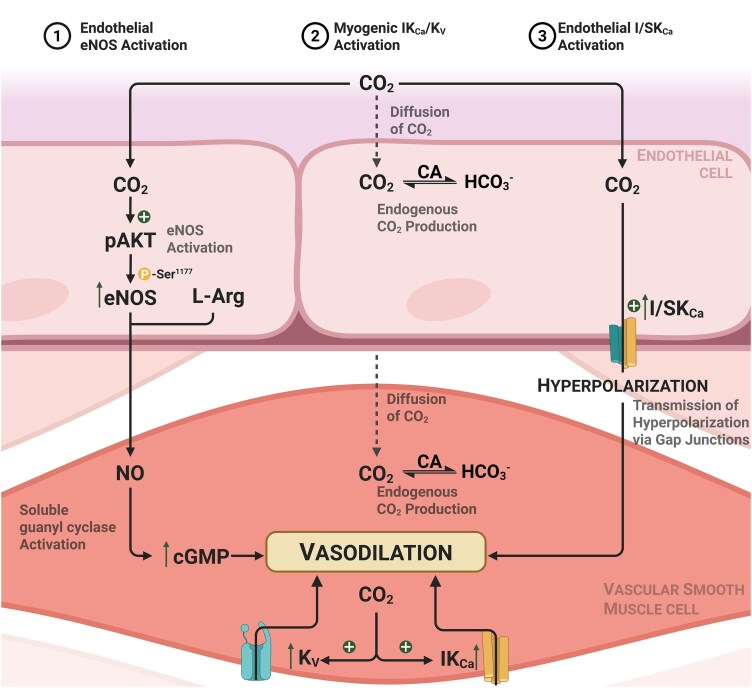

**Time of primary review: 92 days**

## 1. Introduction

Vascular dysfunction is a hallmark of cardiovascular disease, diabetes, sepsis, and autoimmune diseases. It features endothelial and/or myogenic dysfunction with complex local and systemic regulatory circuits. Gasotransmitters, such as nitric oxide (NO), carbon monoxide (CO), hydrogen sulphide (H_2_S), azanone (HNO), and carbon dioxide (CO_2_) are unique among the many vasoactive substances as they easily diffuse through cell membranes and immediately partake in cell signalling.^1^ The vasoactive effects of CO_2_ have been known since the 1930s and are used to treat systemic vascular diseases and diabetic ulcers.^2^ Recently, there has been considerable social media hype on transdermal carboxytherapy for skin rejuvenation and scar healing.

Clinically, CO_2_’s perhaps best-implemented actions are those in the cerebral vasculature, where partial CO_2_ pressure acts as a vasoregulatory sensor of cerebral blood flow and is widely used in intensive care units to treat increased intracranial pressure and cerebral ischaemia.^3^ It also regulates mesenteric and pulmonary vascular tone,^4,5^ with actions ranging from vasodilation to vasoconstriction and mechanisms exercised by partial pressure, NO, pH, or possibly direct CO_2_ effects.^6,7^ CO_2_ levels, transport, and disposal are regulated by carbonic anhydrases (CA), of which several are expressed in vascular endothelial and smooth muscle cells.^8^ Several CA inhibitors induce vasodilation in the cerebral and retinal circulations by not entirely understood mechanisms,^9^ and have been approved for glaucoma, congestive heart failure, idiopathic intracranial hypertension, and acute altitude sickness.^10^

Near-infrared spectroscopy (NIRS) is a non-invasive method to enable monitoring of local tissue oxygenation changes and haemoglobin dynamics, and has been widely used to assess microvascular and perfusion-related physiology.^11^ NIRS has previously been applied to track oxygenation changes during CO_2_ reactivity paradigms, particularly in cerebrovascular research.^12^ In our study, we have unveiled several intertwined effects of endogenous and exogenous CO_2_ on the vasculature that depend on CAs, identified CO_2_ as an endothelium-derived hyperpolarization factor (EDHF)-like vasodilator, linked its action to vascular disease, and designed a technical device to diagnose clinically relevant vascular dysfunction in humans.

## 2. Methods

### 2.1 Study design

The current work presents two sets of different experiments. The first set consisted of an exploratory *ex-vivo* analysis to investigate the effects of CO_2_ on the vasculature. Here, vasoactive CO_2_ effects were tested in arteries of healthy C57BL/6J (BL6) mice, hypertensive apolipoprotein E-deficient mice, and soluble guanylyl cyclase (sGC) knockout mice in a small vessel myograph with and without intervening in endothelium- and/or vascular smooth muscle-mediated vasodilation to unravel the mechanism behind CO_2_ induced vasodilation.

The second set of experiments aimed to apply the initial findings to a diagnostic tool suitable for clinics. Here, CO_2_-based near-infrared spectroscopy (NIRS-CO_2_) was developed to assess the vasoreactivity of skin microcirculation to CO_2_ in healthy individuals and patients with peripheral artery disease (PAD) and coronary artery disease and compare it with their flow-mediated dilation (FMD). These series are reported in accordance with the STROBE (Strengthening the Reporting of Observational Studies in Epidemiology) guidelines for cross-sectional studies.

As this was an exploratory study, no sample size calculation was performed. However, the sample size was determined based on prior experience in murine vasoreactivity studies and in flow-mediated dilation studies in humans. No data or outliers were excluded from the analysis. Each aortic segment originated from an independent mouse and was handled as *n* = 1. Similarly, each NIRS-CO_2_ measurement was performed on an independent subject.

### 2.2 Animal studies

All studies complied with the guidelines of Directive 2010/63/EU of the European Parliament on the protection of animals used for scientific purposes and were approved by the local animal care and ethics committee (LANUV Recklinghausen, Germany) under file numbers 81-02.04.2021.A012 and 81-02.04.2022.A055. Studies were conducted with male BL6 and apolipoprotein E-deficient (ApoE^−/−^) mice (C57BL/6J background) or α1-GC^−/−^ mice (procured from Dr. Evanthia Mergia, Institute for Pharmacology and Toxicology, Ruhr University Bochum). Unless otherwise specified, mice were aged 15–20 weeks, fed a normal chow diet, and had free access to drinking water. For hypertension studies, 15-week-old ApoE^−/−^ mice were fed a Western diet (0.15% cholesterol, 43% kCal fat, 20% kCal carbohydrates) for 5 weeks. After the first week, a subcutaneous osmotic pump containing Angiotensin II (AngII, 1000 ng/kg/min) was implanted after adequate analgesia with Carprofen (5 mg/kg, subcutaneously, 30 min prior to the surgery and on the first two days after surgery), by which hypertension was induced for the following 4 weeks.^13^ All mouse experiments were performed per the European Convention for the Protection of Vertebrate Animals Used for Experimental and Other Scientific Purposes (Council of Europe Treaty Series No. 123).

### 2.3 Human studies

This study included three observational, cross-sectional sub-studies using NIRS in humans. All procedures were conducted in accordance with the Declaration of Helsinki and approved by the local ethics committees. Prior to participation, all individuals provided both verbal and written informed consent.

The first cohort was exploratory and aimed to compare patients with peripheral arterial disease (PAD) treated at the Department of Vascular and Endovascular Surgery, University Hospital Essen (PAD group; *n* = 18) with age-matched controls without PAD (AMC group; *n* = 17). In the second validation set of analyses, we benchmarked NIRS to flow-mediated dilation (FMD) as measured by ultrasound in humans with coronary artery disease (*n* = 10, CAD) and without (*n* = 9). In a third set of experiments, we tested whether the prototype is sensitive to vascular alterations preceding overt clinical disease by assessing young participants with (*n* = 11) and without cardiovascular risk factors (*n* = 24). Young participants were classified as risk-factor-positive if any of the following applied: (i) positive family history of cardiovascular disease, (ii) type 2 diabetes mellitus, (iii) current smoking with cumulative exposure >1 pack-year (PY), (iv) LDL-cholesterol >190 mg/dL, or (v) hypertension (documented elevated blood pressure and/or antihypertensive treatment). Young participants were classified as risk-factor-negative if none of these criteria were met (threshold for risk-factor-negative smokers ≤0.5 PY); former smokers were categorized as young participants without risk factors if cumulative exposure was <5 PY. The first (PAD vs. AMC) and third (young participants ± cardiovascular risk factors) human cohorts were approved by the Ethics Committee at the Faculty of Medicine of the University of Duisburg-Essen under study number 18-8053-BO. The second cohort (participants with and without CAD) was performed in the Cardiology Department of the University Hospital Düsseldorf and approved by the Ethics Committee at the Medical Faculty of Heinrich Heine University Düsseldorf under study number ID2017034183–5903R. All humans received detailed behavioural instructions in accordance with the FMD guidelines prior to measurement.

### 2.4 CO_2_ enrichment

Krebs–Henseleit buffer (KH-buffer: 118 mM NaCl, 4.7 mM KCl, 0.8 mM MgSO_4_, 1.2 mM KH_2_PO_4_, 25 mM NaHCO_3_, 3.33 mM CaCl_2_, and 10 mM d-Glucose) for aortic rings assay was enriched using a water-carbonating device (SodaStream Crystal, SodaStream Inc., Frankfurt am Main, Germany), resulting in a CO_2_ concentration of 7 g/L. Most experiments were conducted in a 1:10 dilution, resulting in a CO_2_ concentration of 0.7 g/L (pCO_2_ 156 mmHg, pH 6.74). CO_2_-enriched solutions were prepared directly prior to the experimental setup. For human experiments, tap water was carbonated using the water-carbonating device. Using the titration method, we estimated a CO_2_ content of 4.2–5.8 g/L (pH 4.9–5.5). Easy-to-use indicator tubes (Gastec Corporation, Kanagawa, Japan) confirmed a CO_2_ content of 4.2–4.9 g/L.

### 2.5 Near-infrared spectroscopy-CO_2_ in humans

NIRS-CO_2_ was performed as follows. Humans placed their left forearm in a custom-made water bath case (*Figure 4D*). The arm was fixed with straps to minimize movement and muscle tension during the measurement. A NIRS probe (OxyMon, Artinis Medical Systems, Elst, The Netherlands) to measure oxyhaemoglobin (HbO_2_) and deoxyhaemoglobin (HHb) was attached to the skin of the forearm by an individually designed holder (*Figure 4D*). The holder had openings for two optodes 1 cm apart that were taped to the skin to allow light skin contact without compressing the subcutaneous tissue. The NIRS device was calibrated at the beginning of each session. For baseline measurements and adaptation, the forearm was entirely covered with 5–6 litres of 32°C tap water (pH 7.85) for 5 min. Then, the tap water was rapidly exchanged for the same volume of CO_2_-enriched water (32°C, pH 4.9). Changes in HbO_2_ and HHb-concentrations were registered by OxySoft software (Artinis Medical Systems, Elst, The Netherlands). After the water exchange, the recording was reset to compensate for the induced artefacts. Several variables were derived from the configurational changes between the HbO_2_ and HHb curves, as illustrated in the representative tracings in Supplementary material online, Figure  *S1*. During establishment, any effects of pH were excluded by comparing the humans’ reaction to CO_2_-enriched tap water in one forearm and tap water adjusted to pH 4.9 in the other. The CO_2_-enriched water provoked the typical changes in the HbO_2_ and HHb recordings (see Supplementary material online, Figure  *S2A*), whereas acidic water did not (see Supplementary material online, Figure  *S2B*).

### 2.6 Flow-mediated dilation for NIRS validation

FMD was measured as previously described at the contralateral arm used for NIRS.^14^ Briefly, the vessel diameter of the radial artery (RA) was measured using a 12 MHz transducer (Vivid I, GE Healthcare, Chicago, IL, USA). Following baseline measurement, a sphygmomanometer cuff was inflated to 200 mmHg for 5 min to achieve complete arterial occlusion and then deflated. The RA diameter was measured at 0, 20, 40, 60, and 80 s thereafter. Analysis was performed using an automatic edge-detection software (Brachial Analyzer, Medical Imaging Applications, Iowa City, Iowa, USA). FMD was calculated as the maximal relative diameter gain following reperfusion relative to baseline.

### 2.7 Vasoreactivity studies using wire myography

Vasoreactivity studies were performed using a previously described protocol with some modifications.^15^ Mice were sacrificed under isoflurane anaesthesia (5% via inhalation in an induction chamber). Once unconsciousness was achieved, the absence of the pedal reflex was confirmed to ensure adequate depth of anaesthesia and prevent discomfort. Euthanasia was performed by cardiac puncture. The heart was then excised, and the thoracic aorta was isolated from adjacent adipose tissue and cut into rings. These were mounted between two 20 µm thick wires in chambers of a wire myograph (Multi Myograph 620 M; Danish Myo Technology A/S, Hinnerup, Denmark) containing 5 mL of KH buffer and continuously gassed with a mixture containing 5% CO_2_ and 95% O_2_. The rings were gradually stretched to a tension of 9.8 mN for 1 h for calibration. After equilibration, the rings were contracted with KCl (80 mM) and phenylephrine (Phe, 10 µM Sigma-Aldrich), and the endothelial function was tested by increasing concentrations of acetylcholine (Ach, 100 pM–10 µM, Sigma-Aldrich, Saint Louis, Missouri, USA) in the presence of indomethacin (Indo, 10 µM; Sigma-Aldrich, Saint Louis, Missouri, USA). The effects of CO_2_ were tested by replacing the perfusion buffer with CO_2_-enriched (0.07 g/L–7 g/L) KH buffer containing the indicated substances. To exclude a pH-dependent vasodilatory component, thoracic aortic rings were exposed to acidified KH buffer (pH 6.74), chosen to match the pH measured in CO_2_-enriched KH buffer by blood gas analysis. Acidification alone did not induce any relevant dilation (see Supplementary material online, Figure  *S2C*). In some experiments, Nω-Nitro-L-arginine methyl ester (L-NAME, 100 µM; Sigma-Aldrich, Saint Louis, Missouri, USA) or 1H-Oxadiazolo[4,3-a]quinoxalin-1-one (ODQ, 10 µM; TOCRIS, Bristol, UK) were applied for a minimum of 15 min before adding Phe. The effectiveness of L-NAME and ODQ was tested by applying Ach (10 µM) and sodium nitroprusside (10 pM–30 µM; Sigma-Aldrich, Saint Louis, Missouri, USA) to precontracted rings. The vascular responses to CO_2_ were calculated as per cent change vs. baseline.

### 2.8 Western blotting

Western blotting was performed with human umbilical vein endothelial cells (HUVECs) and mouse thoracic aortic segments. After cultivation in endothelial cell medium (containing 0.5 mL VEGF, 0.5 mL ECGS, 0.5 mL Heparin, 0.5 mL EGF, 0.5 mL Hydrocortisone, 5.0 mL Antibiotic-Antimycotic Solution, 25.0 mL FBS), HUVECs were treated with medium replacement by KH-buffer with and without CO_2_ enrichment for 30 s. Mouse thoracic aortic segments were isolated and pre-incubated with Phe in KH buffer for 10 min at 37°C to induce contraction. Subsequently, aortic segments were exposed to CO_2_-enriched KH buffer or KH buffer alone as a control for 1 min.

After stimulation, total protein was isolated in RIPA Buffer + Halt™ Protease- und Phosphatase-Inhibitor-Cocktail (Thermo Scientific, Waltham, Massachusetts, USA), followed by a 30-min incubation on ice. Aortic tissue samples were homogenized using a pestle homogenizer prior to incubation. Cell and tissue lysates were cleared by centrifugation at 14 000 g at 4°C for 10 min, and 20 μg of protein was subjected to SDS-PAGE and transferred to PVDF membranes. Target protein detection was performed after blocking in 5% non-fat milk in PBS + Tween20 for 1 h before incubation with primary antibodies (eNOS #32027, Cell Signaling, diluted 1:1000, p-eNOS #9571, Cell Signaling, diluted 1:1000, and #p-Akt 9271, Cell Signaling, diluted 1:500, β-Tubulin #2146, Cell Signaling, diluted 1:1000, Vinculin #4650, Cell Signaling, diluted 1:1000) at 4°C overnight. Afterwards, blots were washed three times, followed by a 1-h incubation with a secondary antibody (Goat Anti-Rabbit IgG Antibody (H + L), Peroxidase (PI-1000-1), Vector Laboratories, diluted 1:5000). Signals were visualized using an enhanced chemiluminescence solution (Immobilon^®^ Forte Western HRP Substrate no. WBLUF0100; Millipore) and detected using ChemiDoc XRS + Imaging System (BioRad, Hercules, California, USA).

### 2.9 Quantitative PCR

RNA was isolated from VSMCs and aorta using the innuPREP RNA Mini Kit (Analytik Jena, Jena, Germany). Synthesis of cDNA (200 ng) was performed by using the Revert Aid First Strand cDNA Synthesis Kit (Thermo Fisher Scientific, Waltham, USA). Gene expression was quantified by quantitative PCR (qPCR) via SYBR Green (Bio-Rad Laboratories, Hercules, USA). Used primer sequences are listed in the data supplement (see Supplementary material online, *Table S1*). As an internal control for normalization, GAPDH was used.

### 2.10 Statistics

Unless otherwise specified, data are shown as mean ± standard error of the mean (SEM). Statistical significances between two groups of continuous variables were determined using an unpaired *t*-test (for normally distributed data) or Mann–Whitney U test (for non-normally distributed data). Normal distribution was assessed by Shapiro–Wilk tests. Ordinal data was assessed using Fisher’s exact test. Statistical comparisons between more groups were assessed by one-way ANOVA. Dose–response curves measured within the vasoreactivity studies were compared using two-way ANOVA with Šídák’s multiple comparisons test. The NIRS-CO_2_ variable with the strongest predictive value for identifying humans with established PAD was determined by comparing the area under the receiver operating characteristic curve (AUC-ROC) and through single logistic regression. The metric with the highest accuracy was then selected as the sole NIRS-CO_2_ predictor for subsequent multivariable (mutually adjusted) logistic regression, adjusting for clinically available covariates (age, sex, BMI, systolic blood pressure, LDL-cholesterol, and heart rate). Results are reported as odds ratios (ORs) with 95% confidence intervals (CIs). The relationship between time-to-intersection (TTI) and FMD was assessed using the Pearson correlation. For all analyses, statistical significance was assumed when *P* ≤ 0.05. All analyses were performed in GraphPad Prism 9 (GraphPad, La Jolla, CA, USA).

## 3. Results

### 3.1 CO_2_ induces vasodilation by two independent mechanisms: an endothelium-reliant through eNOS/NO/sGC and a vascular smooth muscle cell (VSMC)-dependent through IK_Ca_/K_V_

**Figure 1:**
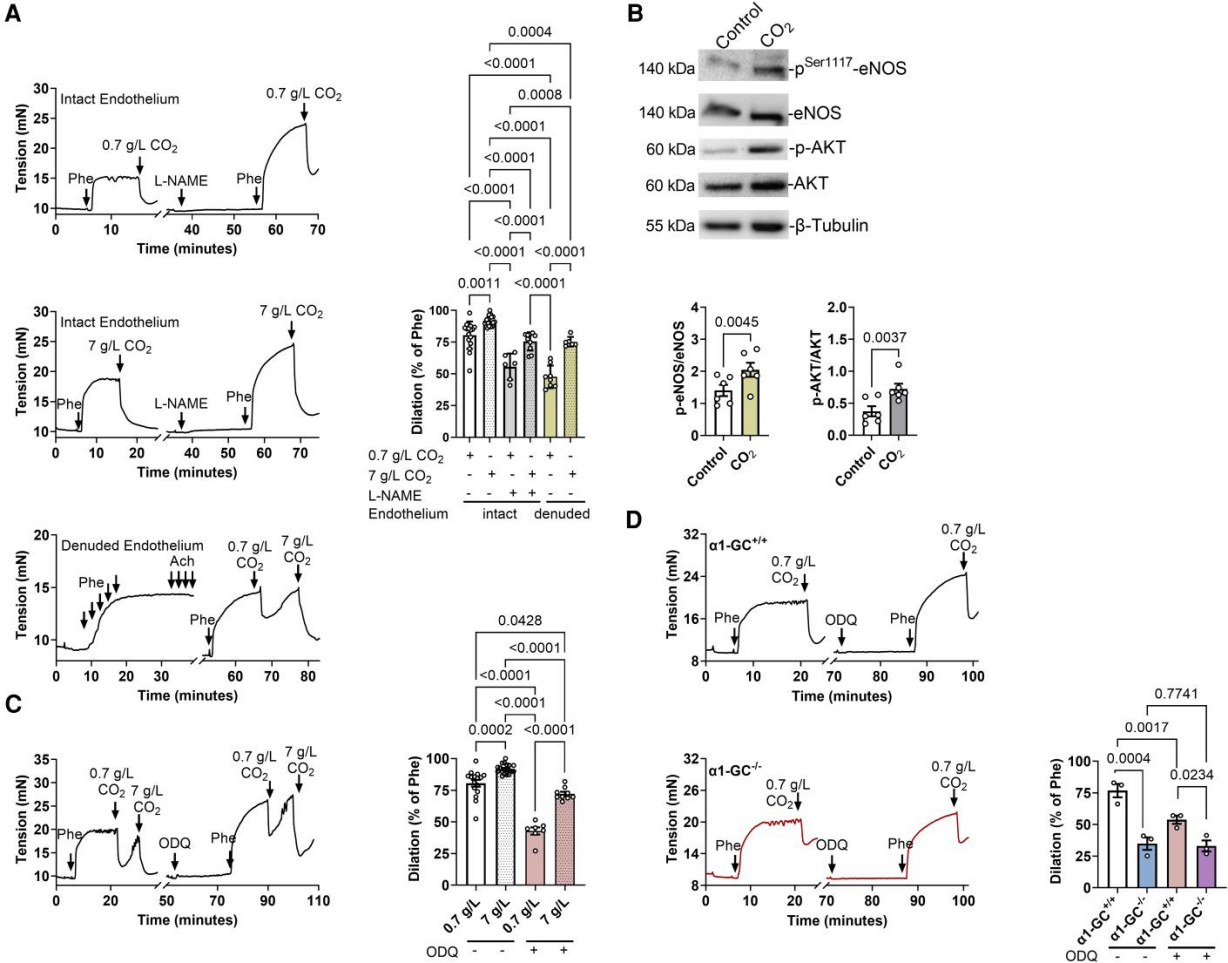
CO_2_ induces vasodilation partly through eNOS/sGC activation. (*A*) Representative tracings and summary quantification of vasodilator responses to CO_2_ (0.7 g/L and 7 g/L) in native aortic rings, after eNOS inhibition with L-NAME (100 µM, 20 min), and after endothelial denudation. *n_CO2_*  _0.7g/L_ = 16, *n_CO2_*  _7g/L_ = 19, *n_CO2_*  _0.7g/L_  _+_  _LNAME_ = 6, *n_CO2_*  _7g/L_  _+_  _LNAME_ = 10, *n_CO2_*  _0.7g/L_  _+_  _Denuded_ = 8, *n_CO2_*  _7g/L_  _+_  _Denuded_ = 6. Data are mean ± SEM. Statistical analyses included one-way ANOVA; *P*-values shown above compared bars. (*B*) Representative immunoblots of phospho-eNOS (Ser1177), total eNOS, phospho-AKT, total AKT, and β-tubulin (loading control) in HUVECs exposed to control KH buffer (Control) or CO_2_-enriched KH buffer (7 g/L). Quantification shows the phospho/total ratios of eNOS and AKT. Dots represent individual biological replicates. Data are mean ± SEM. Statistical analyses included paired *t*-test. *P*-values shown above compared bars. (*C*) CO_2_ (0.7 g/L and 7 g/L) induced dilation in native aortic rings and rings preincubated with the GC-inhibitor ODQ (10 µM, 20 min). *n_CO2_*  _0.7g/L_ = 16, *n_CO2_*  _7g/L_ = 19, *n_CO2_*  _0.7g/L_  _+_  _ODQ_ = 7, *n_CO2_*  _7g/L_  _+_  _ODQ_ = 9. The data points for the native responses to 0.7 g/L and 7 g/L CO_2_ are the same as those shown in panel A. Data are mean ± SEM. One-way ANOVA; *P*-values shown above compared bars. (*D*) CO_2_ (0.7 g/L and 7 g/L) induced dilation in aortic rings from α1-GC^+/+^ and α1-GC^−/−^ mice before and after preincubation with ODQ (10 µM, 20 min). n_α1-GC_^+/+^ = 3, n_α1-GC_^−/−^ = 3. Data are mean ± SEM. Statistical analyses included two-way ANOVA followed by Šídák’s multiple comparisons test; *P*-values shown above compared bars.

CO_2_-enriched KH buffer potently induced vasorelaxation in mouse aortic thoracic rings precontracted with 10 µM Phe in a concentration-dependent manner (*Figure 1A* and Supplementary material online, Figure  *S3B*). Preincubation with the endothelial nitric oxide synthase (eNOS) inhibitor L-NAME reduced CO_2_-mediated vasorelaxation to ∼50% (*Figure 1A*) while completely abolishing vasodilation by Ach (see Supplementary material online, Figure  *S3A*). CO_2_ also induced Ser1177-eNOS phosphorylation and its upstream kinase Akt at Ser473 in a time-dependent manner in endothelial cells (*Figure 1B* and Supplementary material online, Figure  *S3C*) and isolated mice aorta (see Supplementary material online, Figure  *S3D*). Endothelial denudation reduced CO_2_-induced vasodilation, similar to L-NAME (*Figure 1A*).

To explore the nature of the remaining 50%, we hypothesized that CO_2_ may directly activate the soluble guanylyl cyclase (sGC) in a manner similar to CO.^16^ However, the sGC inhibitor ODQ inhibited CO_2_ vasodilation to the same 50% as L-NAME (*Figure 1C*) at concentrations that completely inhibited vasodilation by the NO-donor sodium nitroprusside (SNP, Supplementary material online, Figure  *S4A*). The CO_2_ effect was not further impaired in α1-GC^−/−^ aorta, where CO_2_-vasodilation was also reduced by ∼50% compared to α1-GC^+/+^ (*Figure 1D*). α1-GC^−/−^ aorta had the expected impaired response to Ach^17^ (see Supplementary material online, Figure  *S4B*), while endothelium-independent dilation as a response to SNP is known to be preserved.^17^ GUCY1A2 was robustly expressed in VSMCs and isolated mouse aorta, but was not detectable in HUVECs (see Supplementary material online, Figure  *S4C*). Our data suggested that half of CO_2_’s vasodilatory effect was mediated by eNOS/NO/sGC, rather than acting directly on sGC.

**Figure 2:**
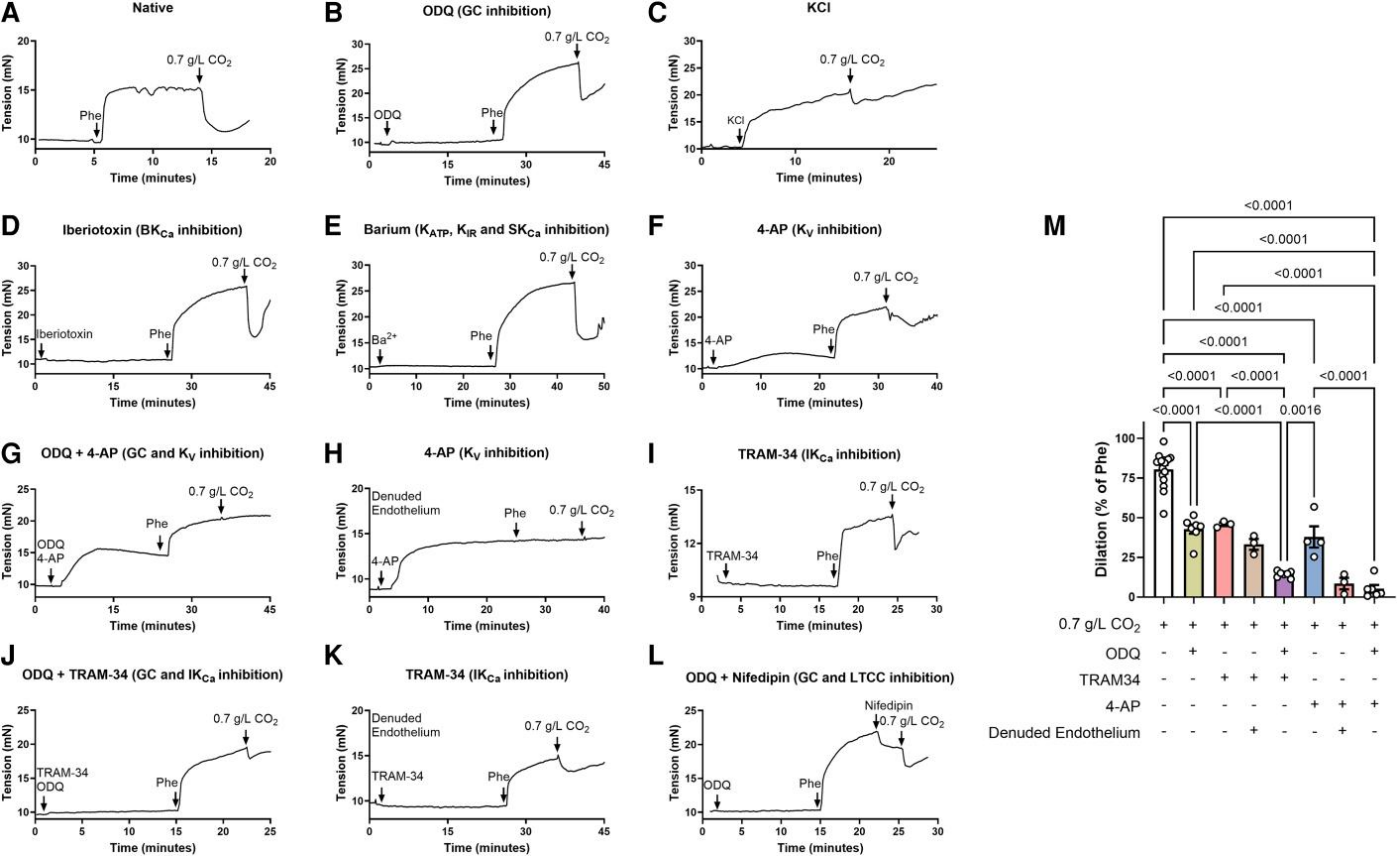
The endothelium-independent component of CO_2_-induced vasodilation is mediated by IK_Ca_ and K_V_. Representative tracing of the vasodilator responses to CO_2_ (0.7 g/L) in (*A*) native aortic rings, (*B*) preincubation with the GC-inhibitor ODQ (10 µM, 20 min), (*C*) preincubation with high concentration KCl (80 mM), (*D*) preincubation with Iberiotoxin (100 nM, 20 min), (*E*) preincubation with the Ba^2+^ (100 µM, 20 min), (*F*) preincubation with 4-Aminopyridine (4-AP, 10 mM, 20 min), (*G*) preincubation with ODQ (10 µM, 20 min) and 4-AP (10 mM, 20 min), (*H*) preincubation with 4-AP (10 mM, 20 min) and denuded endothelium, (*I*) preincubation with TRAM-34 (10 µM, 20 min), (*J*) preincubation with ODQ (10 µM, 20 min) and TRAM-34 (10 µM, 20 min), (*K*) preincubation with TRAM-34 (10 µM, 20 min) and with denuded endothelium, (*L*) preincubation with ODQ (10 µM, 20 min) and nifedipine (10 nM). (*M*) Quantification of the maximal dilation amplitude induced by 0.7 g/L CO_2_ in *A*, *B*, *F*–*K* (*n_native_* = 16, *n_ODQ_* = 7, *n_TRAM_* = 3, *n_TRAM34_  _+_  _Denuded_* = 3, *n_TRAM34_  _+_  _ODQ_* = 7, *n_4-AP_* = 4, *n_4-AP_  _+_  _Denuded_* = 3, *n_4-AP_  _+_  _ODQ_* = 6). Data points for the response of 0.7 g/L CO_2_ in the absence and presence of ODQ are the same as shown in *Figure 1*. Data are mean ± SEM. One-way ANOVA; *P*-values shown above compared bars.

To still pursue the unaccounted part, we hypothesized that VSMC potassium channels, which are pivotal to vascular tone regulation,^18^ might be involved, prompting us to test their contribution to CO_2_ vasodilation with and without sGC inhibition and endothelial denudation, respectively (for comparison, the response without inhibitors is shown in *Figure 2A* and the response in the presence of ODQ in *Figure 2B*). In line with this hypothesis, high extracellular K^+^ (80 mM KCl) markedly blunted CO_2_-induced vasodilation (*Figure 2C*). Thus, we applied: Iberiotoxin (100 nM) for large-conductance calcium-activated potassium channels (BK_Ca_),^19^ Ba^2+^ (100 µM) for ATP-sensitive potassium channels (K_ATP_), inward rectifier potassium channels (K_IR_), and small-conductance calcium-activated potassium channels (SK_Ca_),^20^ TRAM-34 (10 µM) for intermediate-conductance calcium-activated potassium channels (IK_Ca_),^21^ and 4-Aminopyridine (4-AP; 10 mM) for K_V_.^22^ We first excluded BK_Ca_, SK_Ca_, K_IR_, and K_ATP_ as iberiotoxin and Ba^2+^ had no effect on CO_2_ vasodilation (*Figure 2D* and *E*). We concluded that K_V_, IK_Ca_, or a combination of both must be involved. Indeed, vasodilation was decreased by ∼50% in the presence of 4-AP in the absence of ODQ (*Figure 2F*) and completely abolished in its presence (*Figure 2G*) and after endothelial denudation (*Figure 2H*), respectively. TRAM-34 also inhibited vasodilation by ∼50% in the absence of ODQ (*Figure 2I*), which, again, was almost completely lost in the presence of ODQ (*Figure 2J*) or after endothelial denudation (*Figure 2K*). Since voltage-gated calcium channels, such as the L-type calcium channel (LTCC), substantially contribute to the intracellular Ca^2+^ enrichment necessary for vasocontraction by calcium-activated potassium channels like IK_Ca_,^23^ we used the LTCC inhibitor nifedipine (10 nM) in addition to ODQ and, indeed, observed a clear decrease in CO_2_-vasodilation (*Figure 2L*). *Figure 2M* summarizes the quantification of CO_2_-induced vasodilation in the presence of inhibitors relevant to the identified pathways. Altogether, our data suggested that K_V_ and IK_Ca_ were responsible for endothelium-independent, VSMC-dependent dilation by CO_2_.

### 3.2 Endogenous CO_2_ is an EDHF-like vasodilator

**Figure 3:**
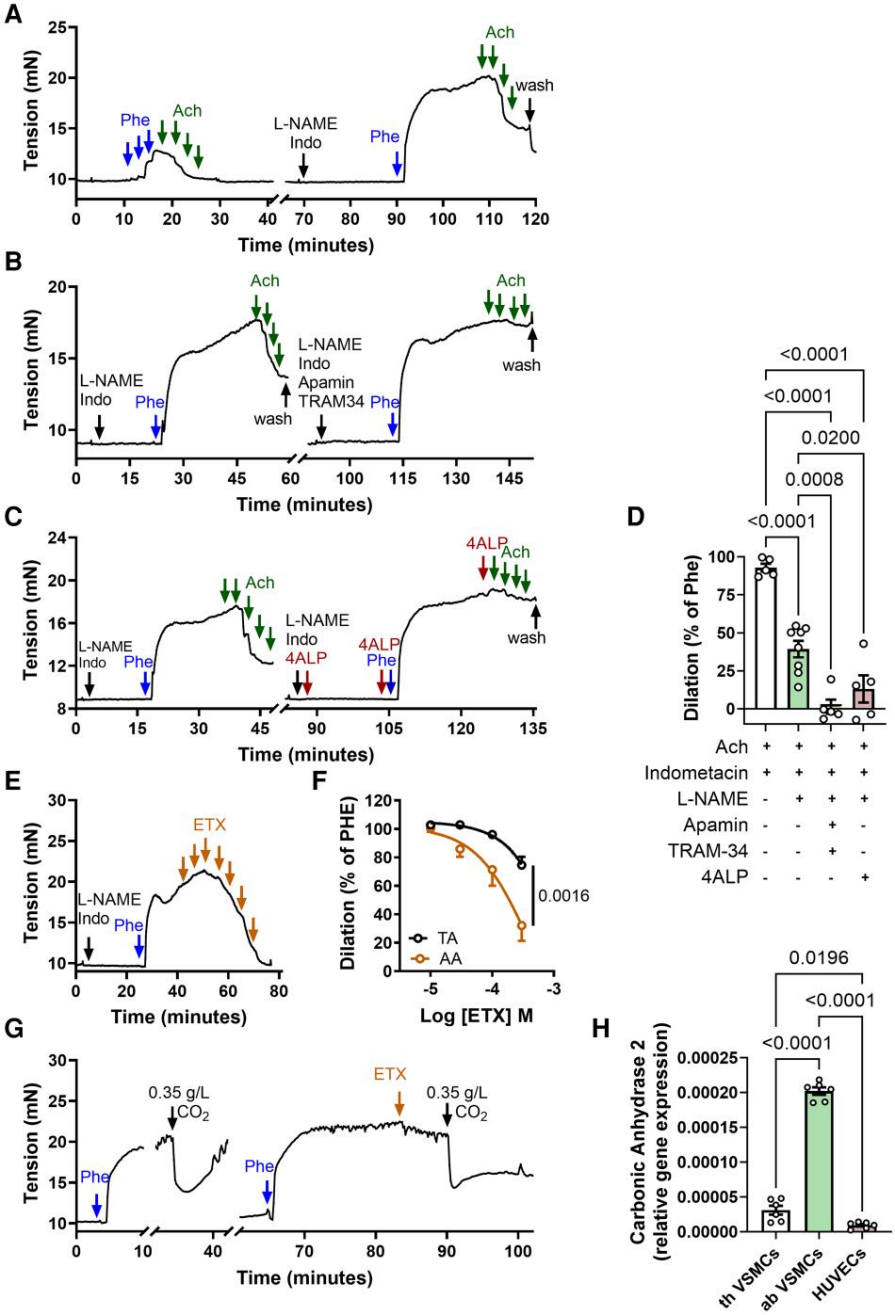
Endogenous CO_2_ is an EDHF-like vasodilator. (*A*) Ach-induced vasodilation in the most distal part of the abdominal aorta was inhibited only by half through L-NAME (100 µM, 20 min) and indomethacin (Indo, 10 µM, 20 min) and (*B*) completely abolished by Apamin (0.5 µM, 20 min) and TRAM-34 (10 µM, 20 min) demonstrating an EDHF-responsive site. (*C*) Abolished EDHF action in the presence of the carbonic anhydrase activator 4-Amino-L-Phenylalanine (4ALP) (30 µM). (*D*) Ach-mediated dilation in abdominal aorta in native segments (*n* = 5), segments treated with L-NAME and Indo (*n* = 8), segments treated with L-NAME, Indo, Apamin and TRAM-34 (*n* = 5), and segments treated with L-NAME, Indo, and 4-ALP (*n* = 5). Data are mean ± S.E.M. One-way ANOVA; *P*-values shown above compared bars. (*E*) Direct vasodilation by the carbonic anhydrase inhibitor Ethoxzolamide (ETX) (3 µM–1 mM) in abdominal aortic rings preincubated with L-NAME (100 µM, 20 min) and Indo (10 µM, 20 min) and (*F*) quantification of the dose-dependent ETX response in abdominal vs. thoracic aortic (*n* = 3) segments showing a more pronounced effect in the abdominal aorta (*n* = 3). Data are mean ± SEM. Two-way ANOVA with RM; *P*-values shown above compared bars. (*G*) ETX at concentrations with no effect (1 µM) substantially prolonged vasodilation by exogenous CO_2_ in a reduced concentration on thoracic aortic segments. The curves shown in the left and right part of the graph originate from two distinct aortic segments. (*H*) Relative gene expression of Carbonic anhydrase 2 in isolated VSMC from thoracic (th VSMCs) and abdominal (ab VSMCs) aorta and from HUVECs, showing markedly higher CA2 expression in abdominal VSMCs. n_th VSMC_ = 6, n_ab VSMC_ = 6, n_HUVECs_ = 6. Data are mean ± SEM. One-way ANOVA; *P*-values shown above compared bars.

We then asked if endogenous CO_2_ also induced vasodilation and focused on endothelial IK_Ca_ as a crucial component of endothelium-derived hyperpolarization (EDH), where chemically still largely unknown EDHFs induce vasodilation independently of NO/sGC or prostanoids by activating endothelial SK_Ca_ and IK_Ca_.^24,25^ To test whether endogenous CO_2_ exerts EDHF-like activity, we tested its action in the distal abdominal aorta as an EDHF-responsive site, where Ach-induced vasorelaxation was inhibited only by half through L-NAME/indo (*Figure 3A*) and completely abolished by additional blockade of SK_Ca_ by Apamin and IK_Ca_ by TRAM-34 (*Figure 3B*). Endogenous CO_2_ is continuously produced by metabolic processes, and its levels, transport, and disposal are regulated by CAs.^9^ CA inhibitors such as ethoxzolamide (ETX) induce vasodilation through rather obscure mechanisms,^9^ prompting us to test whether CO_2_ may be involved. Interestingly, stimulating CA by its activator 4-Amino-L-Phenylalanine (4ALP) completely abolished EDH-mediated vasodilation (*Figure 3C*) to an extent comparable to SK_Ca_ and IK_Ca_ blockade with Apamin/TRAM-34 (*Figure 3D*). Conversely, ETX induced dose-dependent vasodilation in the presence of L-NAME/indo (*Figure 3E* and *F*), and this effect persisted after endothelial denudation (see Supplementary material online, Figure  *S4D*), indicating a partially endothelium-independent mechanism. Intriguingly, at concentrations ETX had no effect itself (1 µM), it substantially prolonged vasodilation by exogenous CO_2_ (representative tracing in *Figure 3G*; pooled data, n = 4, in Supplementary material online, Figure  *S4E*), suggesting that inhibiting CA delays CO_2_ degradation, thus extending its vasodilatory action. Consistent with a segment- and cell-type–specific carbonic anhydrase contribution, qPCR analysis of carbonic anhydrase 2 (CA2) expression in HUVECs and in VSMCs of thoracic and abdominal origin showed the highest CA2 levels in abdominal VSMCs, lower expression in thoracic VSMCs, and near-absence in HUVECs (*Figure 3H*). Our functional data thus suggested CO_2_ as an endogenous EDHF-like vasodilator.

### 3.3 CO_2_ responsiveness identifies endothelial and VSMC dysfunction in hypertensive mice

**Figure 4:**
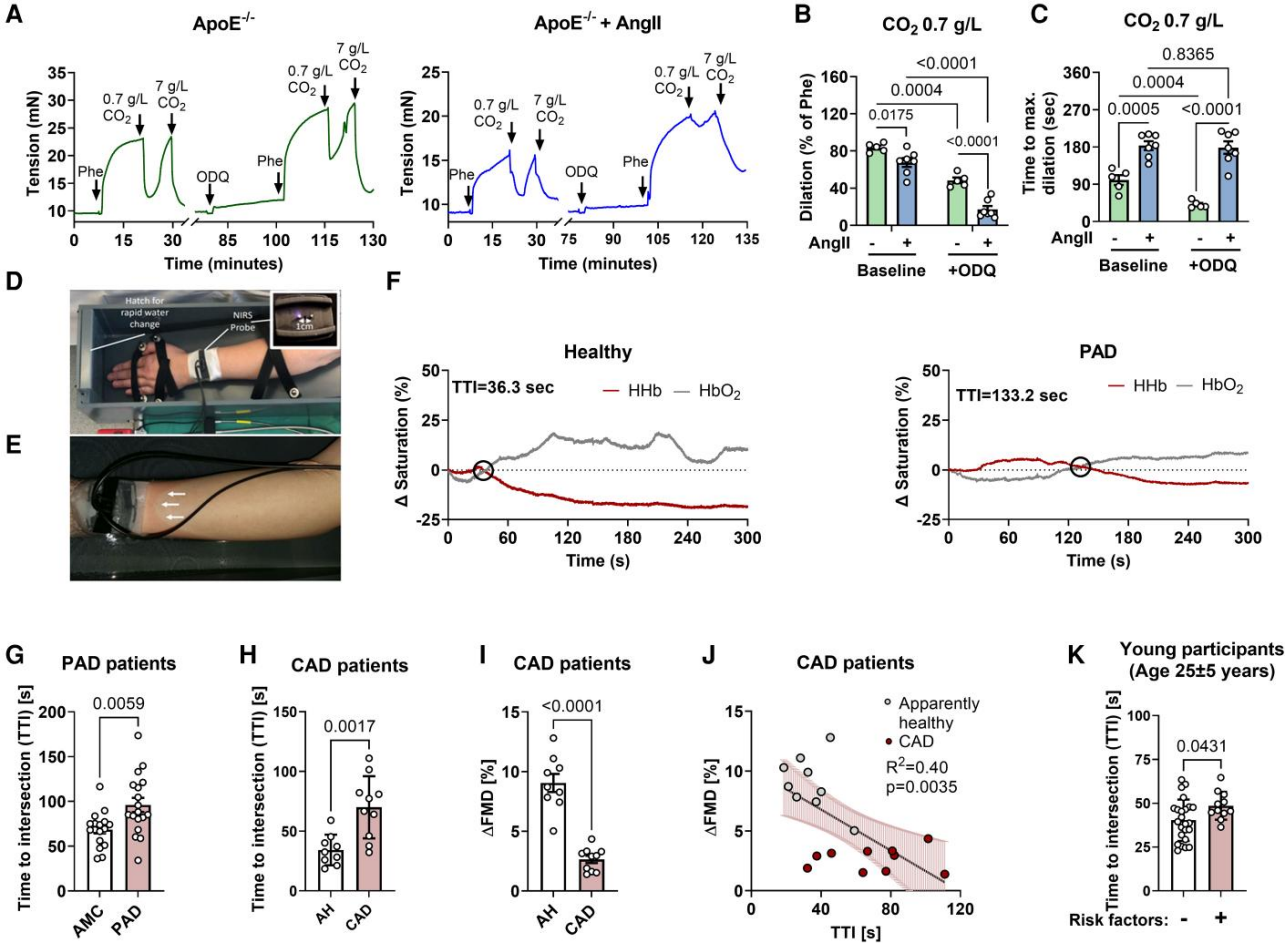
Altered response to CO_2_ in murine and human vascular disease and its detection by NIRS-CO_2_. (*A*) CO_2_-induced dilation in native thoracic aortic rings in the absence and presence of ODQ (10 µM, 20 min) of untreated ApoE^−/−^ mice (green) or hypertensive ApoE^−/−^ mice (blue). (*B*) Maximum dilation amplitude and (*C*) time to maximum dilation in ApoE^−/−^±AngII in the absence and presence of ODQ (*n_ApoE_^−/−^* = 5, *n_ApoE_^−/−^_+AngII_* = 7). Data are mean ± SEM. Two-way ANOVA with RM; *P*-values shown above compared bars. (*D*) Experimental setup: Water bath case with a hatch at one end to release water. The forearm is fixed with straps. The NIRS probe (inset) is attached to the skin with tape. (*E*) Reddish-coloured skin where skin was covered with CO_2_-enriched water, revealing a sharp demarcation (white arrows). (*F*) Representative recordings of HbO_2_ and HHb curves in CO_2_-enriched water in healthy humans and those with peripheral artery disease (PAD). (*G*) TTI is delayed in PAD (*n_PAD_* = 18) vs. age-matched controls (AMC, *n_AMC_* = 17) subjects. (*H* and *I*) TTI is delayed, and FMD is lower in subjects with coronary artery disease (CAD, *n_CAD_* = 10) than in apparently healthy humans (AH, *n_AH_* = 9). (*G–I*): Data are mean ± SEM. unpaired t-tests. *P*-values are indicated above the compared bars. (*J*) Correlation analysis between TTI and FMD in the CAD (*n_CAD_* = 10) and AH (*n_AH_* = 9). Simple linear regression. (*K*) Time-to-intersection is delayed in young participants (age 25 ± 5 years) with (n _+_  _RF_ = 11) vs. those without cardiovascular risk factors (n_-RF_ = 24). Data are mean ± SEM. unpaired *t*-test. *P*-values are indicated above the compared bars.

Defective vasoreactivity has both endothelial and VSMC origins. Since CO_2_-induced vasodilation involved both cell types, we next asked how this response is altered in a model exhibiting combined endothelial and myogenic dysfunction. We therefore studied AngII-treated, hypertensive, hypercholesterolemic ApoE^−/−^ mice, which showed impaired endothelium-dependent relaxation to Ach and a hypercontractile response to Phe (see Supplementary material online, Figure  *S5A*–*C*), while the response to the NO donor SNP was preserved (see Supplementary material online, Figure  *S5D*). While CO_2_ caused the expected vasorelaxation in control ApoE^−/−^ mice, its effect was markedly reduced and delayed (i.e. increased time to maximal dilation) in hypertensive mice in the absence of ODQ and completely abolished in its presence (*Figure 4A–C*). These findings suggest that CO_2_ responses involving both endothelial and VSMC components are blunted, revealing CO_2_'s potential to simultaneously integrate endothelial and myogenic dysfunctional components, a property that we next sought to explore for its diagnostic value in humans.

### 3.4 NIRS-CO_2_ as a diagnostic device for detecting vascular dysfunction in human disease

The gold standard for assessing NO-dependent endothelial function is measuring FMD.^26^ Although the FMD response is elicited in resistance arteries, the actual readout is measured in supplying large conduit arteries. This indirect measurement limits the ability to directly assess arteriole function that coordinates blood pressure, flow, and microcirculation, e.g. in the skin. As cutaneous vasodilation to CO_2_-enriched water has been known for decades,^27^ we constructed a prototype device that employed NIRS to measure skin blood perfusion in response to CO_2_ and used it to compare individuals with humans with peripheral artery disease (PAD) or AMC (see Supplementary material online, *Table S2* for humans’ characteristics). The technique was based on real-time monitoring of oxygenated and deoxygenated haemoglobin (HbO_2_/HHb) as a surrogate for perfusion by exposing a human forearm to CO_2_-enriched water (4.2–4.9 g/L, 32°C; *Figure 4D* and *E*), where HbO_2_ dropped immediately and then began to rise within seconds, eventually reaching a plateau after 1–2 min (*Figure 4F*). Magnitude and changes of this response differed considerably between humans, prompting us to define and evaluate a set of parameters to differentiate between groups. These were TTI between HbO_2_ and HHb curves (representative tracings for healthy individuals and PAD patients are shown in *Figure 4F*), time of mean HbO_2_-HHb distance, time to half-max HbO_2_, time of HbO_2_-x-axis intersection, HbO_2_ upslope, and time of maximum HbO_2_-HHb distance (see Supplementary material online, Figure  *S1*). ROC analysis identified TTI (∼1.4-fold longer in humans with PAD; *Figure 4G*) as the most diagnostically accurate parameter (AUC 0.78, 95% CI: 0.61–0.94, *P* = 0.0048, Supplementary material online, Figure  *S6*), and univariate logistic regression confirmed TTI as the most effective single readout (see Supplementary material online, *Table S3*). To address potential confounding, we fitted a multivariable logistic regression model with PAD status as the outcome, adjusting for clinically available covariates (age, sex, BMI, systolic blood pressure, LDL-C, and heart rate). TTI remained independently associated with PAD (OR 1.056 per second, 95% CI 1.016–1.125; *P* = 0.0286; Supplementary material online, *Table S4*).

We then validated NIRS-CO_2_'s prognostic accuracy and benchmarked it against FMD in an independent cohort of apparently healthy humans and those with CAD (humans’ characteristics in Supplementary material online, *Table S5*). TTI was approximately twice as long in CAD compared to apparently healthy humans (*Figure 4H*), whereas FMD was 3.5-fold lower (*Figure 4I*). In the same individuals, NIRS-CO_2_ and FMD values exhibited a clear negative correlation (*R^2^* = 0.40, *P* = 0.0035, *Figure 4J*). We next examined whether the prototype is sensitive to vascular alterations that precede overt clinical disease by repeating the measurements in young participants (25 ± 5 years) with and without cardiovascular risk factors (see Supplementary material online, *Table S6* for baseline characteristics). Notably, even at this early stage, the risk-factor group exhibited a small yet statistically significant prolongation of TTI (*Figure 4K*). Altogether, NIRS-CO_2_ provides a sensitive, standardized, and non-invasive diagnostic method for detecting abnormal vascular function in humans.

## 4. Discussion

### 4.1 Mechanism of CO_2_ induced vasodilation

The diverse vasoactive CO_2_ effects depend on vessel provenance and involve mechanisms that remain controversially discussed and range from extracellular pH changes to unique CO_2_-intrinsic actions.^28^ The controversy extends to CO_2_'s site of action, with some investigators attributing vasodilation to endothelial NO/sGC signaling^29^ and others demonstrating it in its absence.^3,30^ Our data align with both scenarios as ∼50% of CO_2_-induced vasodilation was mediated through endothelial NO/sGC in conduit vessels, whereas the remaining 50% was exercised by VSMC K_V_ and IK_Ca_ channels. There is no data on CO_2_ explicitly acting on K_V_ and IK_Ca,_ but there is precedence of CO_2_ activating K_IR_^31^ in HeLa cells and even induction of vasodilation through K_ATP_ in cerebral arterioles.^32^ The K_V_ channel is a well-known suppressor of myogenic tone,^18^ and its and that of IK_Ca_ activation promotes K^+^ efflux, membrane hyperpolarization, and closure of voltage-gated calcium channels, leading to vasodilation,^33^ a scenario that explains the loss of vasodilation by K_V_, IK_Ca_, and LTCC inhibitors we observed. We propose that endogenous CO_2_ promotes an EDHF-like component of relaxation in resistance vessels by activating endothelial IK_Ca_. Consistent with this concept, pharmacological inhibition of carbonic anhydrase attenuated vasodilation to a similar extent as combined IK_Ca_/SK_Ca_ blockade, supporting a CO_2_-dependent, K_Ca_-mediated hyperpolarization pathway rather than establishing CO_2_ as a definitive EDHF. This enrols CO_2_ as novel EDHF-like vasodilator alongside *11,12*-epoxyeicosatrienoic acid, H_2_O_2,_ and other gasotransmitters such as H_2_S.^34^ CA inhibitors are known to induce vasodilation through NO/sGC-dependent and -independent as well as endothelium-independent pathways in various species and vascular beds,^35,36^ and CO_2_'s triple mode of vasodilation would reconcile such differences. The vasculature is exposed to systemic CO_2_, but also that generated by vascular cells, and both sources are regulated by local CA activities that dynamically control its vasodilatory potential. In a feedback manner, CO_2_-activated K^+^ channels may also regulate CA activity as K^+^ inhibits mammalian CAs at alkaline pH where CO_2_ hydration is favoured, and K^+^ concentrations as low as 5–10 mM displace H^+^ from CA.^37^ In fact, vasodilation by acetazolamide was demonstrated to depend on K_Ca_ channel activity in human vessels.^36^ We did not observe CO_2_-induced vasodilation during K^+^-induced vasoconstriction, consistent with findings by Mulvany *et al*.^38^ and others who reported that elevated pCO_2_ and vasodilatation were associated with VSMC hyperpolarization through increased K^+^ permeability.^39^ The question of how CO_2_ exactly activated Akt/eNOS in our study is less clear, although there is ample precedent for CO_2_ stimulating various MAPKs, AMPK, and adenylyl cyclase^40,41^ and triggering NO generation and signalling in plants.^42^ Even CO_2_-driven NO generation from nitrite by CA has also been proposed, but also challenged.^9^

### 4.2 NIRS-CO_2_ is a diagnostic tool for the assessment of endothelial function and myogenic tone in humans

Although widely used to assess NO-dependent endothelial function,^26^ FMD is limited to large arteries and remains technically challenging, observer-dependent, and requires specialized training and standardization.^43^ Optical Coherence Tomography Angiography reflects pathological changes in the retinal microvasculature,^44^ whereas Fingertip Peripheral Arterial Tonometry^45^ has similar diagnostic and prognostic value as FMD.^46^ Our NIRS-CO_2_ technique enabled a clear separation of healthy participants from individuals with cardiovascular disease and suggested sensitivity to subtle vascular alterations that may occur before overt dysfunction becomes clinically apparent. However, NIRS-CO_2_ may provide information beyond NO-dependent endothelial dysfunction, as it may also reflect K^+^-channel-dependent myogenic tone, an additional component of vascular dysfunction that is not readily captured by standard vascular function assays and has been implicated in hypertension, heart failure, atherosclerosis, and pulmonary hypertension.^47–49^ Measuring myogenic tone is highly relevant as K_Ca_ dysfunction has been associated with hypertension, diabetes, and chronic kidney disease,^50^ and pharmacological activators of SK_Ca_ and IK_Ca_ have been designed to potentiate EDHF responses and lower blood pressure.^51^ Equally important is assessing myogenic K_V_ activity, as it was reduced in several hypertension models,^52^ caused vasoconstriction in human primary pulmonary hypertension,^53^ and its inhibition induced vasoconstriction in diseased but not control arteries.^54^ NIRS-CO_2_ might provide valuable insight specifically into K_V_- and IK_Ca_-regulated myogenic tone in healthy and diseased human arteries, as hypertensive mouse arteries not only featured a reduced CO_2_ response amplitude but also a remarkable timely delay, which finds its human correlate in the TTI delay in PAD and CAD, ascertaining it as a measurable parameter of myogenic function.

Several limitations should be acknowledged. We did not directly measure endothelial membrane potential changes, and therefore, we frame CO_2_ as an EDHF-like vasodilator based on functional criteria rather than as a definitive EDHF. For NIRS-CO_2_ measurements, residual confounding cannot be excluded, particularly because current smoking was more prevalent among PAD patients and young participants with cardiovascular risk factors, and because the CAD and healthy cohorts used for the FMD comparison were not age-matched. This is especially relevant given that NIRS-derived oxygenation metrics have been shown to decline with increasing age in a large population-based study of the cerebral circulation.^55^ A direct comparison of FMD between the AMC and PAD groups was not performed. In addition, NMD was not assessed; future prospective studies should include NMD to better separate endothelial and smooth muscle contributions. Although TTI and FMD showed a clear inverse correlation when healthy controls and CAD patients were analysed together, after stratification, there was no association between the two variables in the CAD patients (CAD patients: *R*^2^ = 0.006, *P* = 0.87, Supplementary material online, Figure  *S7B*). In the healthy controls, we observed a trend (but without statistical significance; *R*^2^ = 0.14, *P* = 0.32, Supplementary material online, Figure  *S7A*), which may be due to the small sample size. Importantly, this pattern may also be biologically plausible: within a very healthy cohort or a clinically advanced CAD cohort, endothelial function assessed by FMD may cluster in a relatively narrow, low range, making it unsuitable for linear correlation.

In summary, our data support NIRS-CO_2_ as a feasible physiological readout of CO_2_-evoked microvascular reactivity, likely integrating endothelial and myogenic components. In this proof-of-concept setting, NIRS-CO_2_ demonstrated disease-associated alterations, motivating larger, standardized studies to determine its clinical utility across a broader range of vasculopathies, including diabetes-related micro- and macroangiopathy, sepsis-associated vascular dysregulation, and vasospastic or autoimmune conditions. In parallel, further mechanistic work could help uncover CO_2_'s potential for therapeutic modulation in vascular disease.

Translational perspective CO_2_ induces potent dilation by activating the endothelial nitric oxide synthase, endothelial and myogenic calcium-activated potassium channels, as well as smooth muscle cell voltage-gated potassium channels. CO_2_ produced by local carbonic anhydrases in the vasculature mimics the effects of the endothelium-derived hyperpolarizing factor. Cardiovascular alterations lead to the loss of CO_2_-mediated dilation, highlighting the disappearance of intrinsic organ-protective effects in cardiovascular diseases. Therefore, the use of these properties in NIRS-CO_2_ measurements may prove useful for the early detection of vascular dysfunction in cardiovascular, diabetic, and vasospastic angiopathies. Exploring CO_2_’s therapeutic potential could offer novel options in their treatment.

## Supplementary Material

cvag134_Supplementary_Data

## Data Availability

The data underlying this article are available in the article and in its online supplementary material.
