# Supplementary material for: Carbon dioxide is a triple vasodilator
Source: Cardiovasc Res. 2026 Jun 23;122(11):1530–41. doi: 10.1093/cvr/cvag134 (PMC13473971; doi:10.1093/cvr/cvag134)
Supplement: Supplementary file 1 [file cvag134_supplementary_data.zip › Full_Western_Blots (1).docx]

**Western Blots**

The panel below documents the raw western blot used for Fig. 1 and Fig. S3C of the manuscript, including the order of loaded samples, treatment conditions (Krebs-Henseleit solution vs. CO_2_), and duration of exposure to each treatment:

The panel below documents the raw western blot used for Fig. S3D of the manuscript, including the order of loaded samples and treatment conditions (Phe + Krebs-Henseleit solution vs. Phe + CO_2_):
