# Supplementary material for: Carbon dioxide is a triple vasodilator
Source: Cardiovasc Res. 2026 Jun 23;122(11):1530–41. doi: 10.1093/cvr/cvag134 (PMC13473971; doi:10.1093/cvr/cvag134)
Supplement: Supplementary file 1 [file cvag134_supplementary_data.zip › R1_CVR_CO2_SI_black_final.docx]

Carbon dioxide is a triple vasodilator

**- Supplemental Material -**

Dragos A. Duse ^1,2^, Nathalie H. Schröder ^1^, Sotirios Akritidis ^3^, Julia Kälsch ^4^, Dorothe Möllmann ^5^, Martin Schlattjan ^5^, Julia Hoppe ^1^, Evanthia Mergia ^6^, Martin Lainka ^3^, Kurt W. Schmid ^5^, Sylvia Hartmann ^5^, Christoph M. Schaefer ^5^, Finn Wichmann ^5^, Amin Polzin ^2,7^, Ralf Erkens ^2^, Christian Jung ^2,7^, Malte Kelm ^2,7^, Bodo Levkau ^1, 7†^*, Hideo A. Baba ^5†^

**Affiliations:**

^1^ Institute for Molecular Medicine III, University Hospital Düsseldorf, Heinrich Heine University; Düsseldorf, Germany.

^2^ Department of Cardiology, Pulmonology, and Vascular Medicine, Medical Faculty, Heinrich Heine University; Düsseldorf, Germany

^3^ Division of Vascular and Endovascular Surgery, Department of General, Visceral, Vascular and Transplantation Surgery, University of Duisburg-Essen; Essen, Germany

^4^ Department of Gastroenterology and Hepatology, University Duisburg-Essen, Essen, Germany

^5^ Institute of Pathology, University of Duisburg-Essen; Essen, Germany

^6^ Institute of Pharmacology and Toxicology, Ruhr-University Bochum; Bochum, Germany.

^7^ CARID Cardiovascular Research Institute Düsseldorf; Düsseldorf, Germany

^†^ These authors contributed equally to this work

**Supplemental Figures**

**Fig. S1:** **Schematic definition of NIRS-CO_2_-derived response parameters.** Representative HbO_2_ (red) and HHb (gray) curves showing the six parameters used to quantify the temporal characteristics of the CO_2_-evoked response: time to intersection (TTI) of HbO_2_ and HHb, time of mean HbO_2_-HHb distance, time to half-maximal HbO_2_, time of HbO_2_-x-axis intersection, HbO_2_ upslope, and time of maximum HbO_2_-HHb distance.

**Fig. S2:** **The dilatory effects are driven by CO_2_ and not pH.** (A) Left: Recording of HbO_2_ and HHb curves starting with a 5-minute baseline measurement in tap water (pH 7.85), followed by a water exchange with CO_2_-enriched tap water (pH 4.9) and an additional 5-minute recording. The water exchange induces artefacts. Right: The same recording, but with the HbO_2_ and HHb curves reset to zero after the water exchange. Exposure to CO_2_-enriched tap water causes an immediate decrease of HbO_2_, followed by an increase and crosses the HHb curve (=TTI). (B) Left: Recording of HbO_2_ and HHb curves in tap water (pH 7.85). After water exchanging the water with tap water adjusted to pH 4.9, no changes in the HbO_2_ and HHb curves were observed. This suggests that CO_2,_ rather than low pH, is responsible for the specific changes of the HbO_2_ and HHb curves. (C) Extracellular acidification does not elicit vasodilation in precontracted aortic rings. Representative myograph tracing showing isometric tension in thoracic aortic rings. Rings were precontracted with phenylephrine (Phe), followed by exposure to Krebs-Henseleit (KH) buffer adjusted to pH 6.74 (arrow), which did not induce relaxation. The solid black line denotes the mean tension over time; red dotted lines indicate variability (± SEM) across replicates (n=4).

**Fig. S3:** (A) Visual representation of the dilative response to CO_2_ concentrations between 0.07 and 7 g/L. (B) Ach (10 µM) mediated dilation is abolished in thoracic aortic rings precontracted with Phe (10 µM) and preincubated with eNOS-inhibitor L-NAME (100 µM, 20 minutes). (C) Time-dependent effects of CO_2_ on AKT and eNOS phosphorylation in human umbilical vein endothelial cells (HUVECs) were exposed to CO_2_ or time-matched control conditions (Ctrl.) for 30 s, 1, 2, 5, or 10 min, immediately shock-frozen, and analysed by Western blotting. Representative immunoblots show phosphorylated AKT (p-AKT), total AKT (AKT), phosphorylated eNOS at Ser^1177^ (p-eNOS^Ser1177^), total eNOS, and β-tubulin (loading control). Right panels show densitometric quantification of p-AKT normalized to total AKT (p-AKT/AKT) and p-eNOS normalized to total eNOS (p-eNOS/eNOS) at 1, 2, 5, and 10 min; dots represent independent biological replicates and bars depict mean ± SEM. P-values for Ctrl. vs CO_2_ at each time point are indicated in the graphs. (D) Representative immunoblots and quantification of eNOS phosphorylation in isolated thoracic mouse aortic segments. Segments were stimulated with Phe in control Krebs-Henseleit (KH) buffer or in CO_2_-enriched KH buffer (7 g/L). Blots show phospho-eNOS (Ser1177), total eNOS, and vinculin (loading control). Bar graph depicts the p-eNOS/total eNOS ratio (A.U.); dots represent individual samples; data are mean ± SEM.

**Fig. S4:** (A) SNP (10 pM–30 µM) mediated dilation is abolished in thoracic aortic rings precontracted with Phe (10 µM) and preincubated with GC-inhibitor ODQ (10 µM, 20 minutes). (B) Dose-dependent relaxation curve of Ach in thoracic aorta of α1-GC^+/+^ (black) or α1-GC^-/-^ mice (red) in the presence of indomethacin (indo, 10 µM). n_α1-GC_^+/+^=3, n_α1-GC_^-/-^=3. Two-way ANOVA with RM. p-value shown between the compared dose-response curves. (C) Relative GUCY1A2 gene expression is shown for vascular smooth muscle cells (VSMCs), human umbilical vein endothelial cells (HUVECs), and mouse thoracic aortic segments. Open circles represent individual biological replicates; data are mean ± SEM. GUCY1A2 was detectable in VSMCs and aorta but not detected (n.d.) in HUVECs. n_VSMC_=12, n_Aorta_=10. (D) Preserved effect of Ethoxzolamide (ETX, 10 µM – 1 mM) in abdominal aortic segments with denuded endothelium. (E): Pooled myograph tracing of isometric tension in Phe-precontracted mouse aortic rings, expressed as % of maximal Phe contraction. Pre-incubation with Ethoxzolamide (ETX; 1 µM), a concentration that did not affect vascular tone per se, prolonged the CO_2_-evoked relaxation, consistent with carbonic anhydrase inhibition delaying CO_2_ degradation and thereby extending its vasodilatory action. Solid line denotes the mean and dotted lines the SEM; n = 4 vessel segments.

**Fig. S5: Four-week Angiotensin II (AngII) treatment leads to endothelial dysfunction in response to Ach and hypercontractile response to Phe, while the endothelium-independent relaxation remains preserved in cholesterol-fed ApoE^-/-^ mice.** (A-C) Representative tracings and quantification to the dose-dependent response to Ach and Phe (*n_ApoE_^-/-^*=5, *n_ApoE_^-/-^_+AngII_*=7). (D) Quantification of the dose-dependent response to SNP (*n_ApoE_^-/-^*=4, *n_ApoE_^-/-^_+AngII_*=6). Data are mean ± S.E.M. Two-way ANOVA with RM; p-values shown above compared bars.

**Fig. S6: ROC curves for NIRS-CO_2_ parameters (time to intersection (TTI), timepoint of mean HbO_2_-HHb distance, timepoint of half-max HbO_2_, timepoint of HbO_2_-x-axis intersection, HbO_2_ upslope and timepoint of max HbO_2_-HHb distance) and their diagnostic accuracy in distinguishing between humans with peripheral artery disease (PAD) and age-matched controls (AMC).** AUC, 95% CI, and p-values are shown in each curve.

**Fig. S7: Association of NIRS-CO_2_-derived time-to-intersection (TTI) with flow-mediated dilation (FMD) in healthy individuals (A) and patients with coronary artery disease (CAD) (B). This figure shows the group-stratified analyses corresponding to the combined analysis presented in Fig. 4J.** Solid lines indicate linear regression fits with 95% confidence intervals. No significant association between TTI and FMD was observed in either group.

**Table S1: Primer sequences used for quantitative PCR**

| **Gene** | **Sequence (5’->3’)** |
| --- | --- |
| Carbonic anhydrase 2 (Ca2) | CTTGCTCCCTTCTTCCTGGA  CAGGTCACACATTCCAGCAG |
| Glyceraldehyde 3-phosphate dehydrogenase (Gapdh) | AGGTCGGTGTGAACGGATTTG  TGTAGACCATGTAGTTGAGGTCA |
| Guanylate cyclase 1 soluble subunit alpha 2 (Gucy1a2) | CCTTGCCTCAGACGATACACA  GCAGTCCGAGTACATTAGCAGT |

**Table S2: Baseline characteristics of humans with peripheral artery disease (PAD, n=18) and age-matched controls (AMC, n=17).** Statistical testing included unpaired t-test (for age, body-mass index and vital parameters) and Fishers exact test (for gender and proportions of cardiovascular risk factors).

|  | **AMC** | **PAD** | **p-value** |
| --- | --- | --- | --- |
| n | 17 | 18 |  |
| Age (years) | 63±6 | 63±5 | 0.92 |
| Gender (male) n, (%) | 7 (41) | 8 (44) | 0.99 |
| BMI (kg/m2) | 27±3 | 26±4 | 0.37 |
| Cardiovascular risk factors |  |  |  |
| Currently smoking n, (%) | 0 (0) | 13 (72) | <0.0001 |
| Hypertension n, (%) | 10 (59) | 14 (78) | 0.29 |
| Dyslipidaemia n, (%) | 11 (65) | 10 (56) | 0.73 |
| Diabetes mellitus n, (%) | 7 (41) | 7 (39) | 0.99 |
| Vital parameters |  |  |  |
| Systolic blood pressure (mmHg) | 132±10 | 129±19 | 0.59 |
| Diastolic blood pressure (mmHg) | 85±7 | 81±11 | 0.17 |
| Heart rate (bpm) | 73±22 | 68±11 | 0.90 |

**Table S3: Univariable logistic regression analyses of NIRS-CO_2_-derived measures associated with peripheral artery disease (PAD) status (exploratory).** Each row represents a separate univariable logistic regression model (one NIRS-CO₂ measure per model). n_AMC_=17, n_PAD_=18 for all variables. p-values calculated for the area under the receiver operating characteristic (ROC) curve, with 95% confidence intervals determined using the Wilson/Brown method.

| **Variable** | **Odds ratios** | **95% CI** | **p-value** |
| --- | --- | --- | --- |
| Time point of HbO_2_- HHb intersection (=TTI) [s] | 1.043 | 1.01 to 1.09 | 0.02 |
| Time point of mean HbO_2_-HHb distance [s] | 1.038 | 1.01 to 1.07 | 0.02 |
| Time point of half-max HbO_2_ [s] | 1.01 | 0.99 to 1.02 | 0.17 |
| Time point of HbO_2_-x-axis intersection [s] | 1.01 | 0.99 to 1.02 | 0.39 |
| HbO_2_ upslope [A.U.] | 0.01 | 4e-025 to 3e+016 | 0.23 |
| Time point of max HbO_2_-HHb distance [s] | 1.001 | 0.99 to 1.01 | 0.86 |

**Table S4:** Adjusted odds ratios from multivariable logistic regression models for PAD status (including TTI and clinical covariates).

| **Multiple logistic regression** | | | |
| --- | --- | --- | --- |
| **Variable** | **Estimate** | **95% CI (profile likelihood)** | **p-value** |
| TTI [s] | 1.056 | 1.01 to 1.13 | **0.03** |
| Age | 0.958 | 0.80 to 1.13 | 0.61 |
| BMI | 0.9233 | 0.69 to 1.21 | 0.56 |
| Sex [1] | 0.2708 | 0.02 to 1.9 | 0.22 |
| SBP | 0.959 | 0.88 to 1.03 | 0.29 |
| LDL-C | 0.9843 | 0.95 to 1.01 | 0.24 |
| HR | 0.963 | 0.86 to 1.04 | 0.42 |

**Table S5: Baseline characteristics of the apparently healthy humans (n=9) and those with coronary artery disease (CAD, n=10).** Statistical testing included unpaired t-test (for body-mass index), Mann-Whitney test (for age), and Fisher's exact test (for gender and proportions of cardiovascular risk factors).

|  | **Apparently healthy** | **CAD** | **p-value** |
| --- | --- | --- | --- |
| n | 9 | 10 |  |
| Age (years) | 29±6 | 82±4 | <0.0001 |
| Gender (male) n, (%) | 7 (78) | 6 (60) | 0.63 |
| BMI (kg/m^2^) | 23±3 | 27±4 | 0.04 |
| Cardiovascular risk factors |  |  |  |
| Currently smoking n, (%) | 0 (0) | 0 (0) | 0.99 |
| Hypertension n, (%) | 2 (22) | 9 (90) | 0.0055 |
| Dyslipidemia n, (%) | 0 (0) | 6 (60) | 0.0108 |
| Diabetes mellitus n, (%) | 0 (0) | 3 (30) | 0.21 |

**Table S6: Baseline characteristics of young participants without cardiovascular risk factors (n=24) and those with cardiovascular risk factors (n=11).** Statistical testing included unpaired t-test (for age, body-mass index, and vital parameters) and Fisher's exact test (for gender and proportions of cardiovascular risk factors).

|  | **Participants without CVRF** | **Participants with CVRF** | **p-value** |
| --- | --- | --- | --- |
| n | 24 | 11 |  |
| Age (years) | 24±5 | 25±4 | 0.81 |
| Gender (male) n, (%) | 10 (42) | 6 (54) | 0.99 |
| BMI (kg/m^2^) | 22±3 | 22±3 | 0.69 |
| Cardiovascular risk factors |  |  |  |
| Currently smoking n, (%) | 0 (0) | 9 (81) | <0.0001 |
| Hypertension n, (%) | 0 (0) | 1 (9) | 0.31 |
| Dyslipidaemia (LDL-C >190 mg/dL) n, (%) | 0 (0) | 0 (0) | 0.99 |
| Diabetes mellitus n, (%) | 0 (0) | 0 (0) | 0.99 |
| Cardiovascular disease history | 0 (0) | 2 (18) | 0.09 |
| Vital parameters |  |  |  |
| Systolic blood pressure (mmHg) | 118±10 | 116±10 | 0.70 |
| Diastolic blood pressure (mmHg) | 73±7 | 74±7 | 0.89 |
| Heart rate (bpm) | 64±10 | 65±6 | 0.67 |
